# Non-invasive physical plasma for preventing radiation dermatitis in breast cancer: study protocol for a phase 3 randomised double-blind placebo-controlled trial (NIPP-RD III)

**DOI:** 10.1186/s13063-025-08806-w

**Published:** 2025-03-19

**Authors:** Cas Stefaan Dejonckheere, Julian Philipp Layer, Gustavo Renato Sarria, Shari Wiegreffe, Andrea Renate Glasmacher, Younèss Nour, Davide Scafa, Thomas Müdder, Teresa Anzböck, Frank Anton Giordano, Matthias Bernhard Stope, Leonard Christopher Schmeel, Eleni Gkika

**Affiliations:** 1 Department of Radiation Oncology, University Hospital Bonn, Venusberg-Campus 1, Bonn, 53127 Germany; 2 Institute of Experimental Oncology, University Hospital Bonn, Bonn, 53127 Germany; 3 Department of Gynaecology and Gynaecological Oncology, University Hospital Bonn, Bonn, 53127 Germany; 4 Department of Radiation Oncology, University Medical Center Mannheim, Mannheim, 68167 Germany; 5 DKFZ-Hector Cancer Institute, University Medical Center Mannheim, Mannheim, 68167 Germany; 6 Department of Gynaecology and Gynaecological Oncology, Physical Plasma Laboratories, University Hospital Bonn, Bonn, 53127 Germany

**Keywords:** Radiation dermatitis, Radiation therapy, Breast cancer, Non-invasive physical plasma, Cold atmospheric plasma, Randomised controlled trial

## Abstract

**Background:**

Radiation dermatitis (RD) is the most common side effect of breast irradiation, yet only few potent preventative and therapeutic options are available. Following encouraging results from a phase 1 and 2 trial on the topical use of non-invasive physical plasma (NIPP), a very well-tolerated physical treatment option to promote tissue regeneration generated from ambient air, we now present the study protocol for a planned phase 3 trial.

**Methods:**

In this randomised double-blind placebo-controlled trial, patients with breast cancer will be randomised (1:1) to receive either 120 s of NIPP or sham treatment with an identical device daily during hypofractionated breast irradiation following breast-conserving surgery. Standard skin care with urea lotion will be applied twice daily to the whole breast by all patients. Acute skin toxicity will be assessed weekly and includes clinician- (CTCAE v5.0) and patient-reported (modified RISRAS), and objective (spectrophotometry) assessments. The trial has started enrolment in the first quarter of 2024 and is projected to recruit 140 patients over 36 months.

**Discussion:**

This randomised controlled trial will recruit a homogeneous patient collective in terms of RD risk and aims to unequivocally establish the impact of NIPP on RD by employing a robust trial design, incorporating both the patient’s perspective and validated objective outcome measures. If the addition of NIPP proves useful, it might reduce both physical and psychological distress caused by RD in numerous breast cancer patients and beyond.

**Trial registration:**

German Clinical Trial Registry DRKS00032560 (January 9th 2024).

## Administrative information

The numbers in curly brackets in this protocol refer to SPIRIT checklist item numbers. The order of the items has been modified to group similar items (see http://www.equator-network.org/reporting-guidelines/spirit-2013-statement-defining-standard-protocol-items-for-clinical-trials/).

**Table:** 

Title {1}	Non-Invasive Physical Plasma for Preventing Radiation Dermatitis in Breast Cancer: Study Protocol for a Phase 3 Randomised Double-Blind Placebo-Controlled Trial (NIPP-RD III)
Trial Registration {2a and 2b}	This trial has been registered prospectively in the German Clinical Trial Registry (DRKS) on January 9th 2024, identifier DRKS00032560.
Protocol Version {3}	Version 1.0 of the protocol from November 9th, 2023.
Funding {4}	This trial is funded by the *Förderinstrument Klinische Studien* of the University Bonn, Germany (grant number 2023 − FKS − 05). The devices are provided free of charge by terraplasma medical (Garching, Germany).
Author Details {5a}	Cas Stefaan Dejonckheere: Department of Radiation Oncology, University Hospital Bonn, 53,127 Bonn, Germany (cas.dejonckheere@ukbonn.de); Julian Philipp Layer: Department of Radiation Oncology, University Hospital Bonn, 53,127 Bonn, Germany and Institute of Experimental Oncology, University Hospital Bonn, 53,127 Bonn, Germany; Gustavo Renato Sarria: Department of Radiation Oncology, University Hospital Bonn, 53,127 Bonn, Germany; Shari Wiegreffe: Department of Radiation Oncology, University Hospital Bonn, 53,127 Bonn, Germany; Andrea Renate Glasmacher: Department of Radiation Oncology, University Hospital Bonn, 53,127 Bonn, Germany; Younèss Nour: Department of Radiation Oncology, University Hospital Bonn, 53,127 Bonn, Germany; Davide Scafa: Department of Radiation Oncology, University Hospital Bonn, 53,127 Bonn, Germany; Thomas Müdder: Department of Radiation Oncology, University Hospital Bonn, 53,127 Bonn, Germany; Teresa Anzböck: Department of Gynaecology and Gynaecological Oncology, University Hospital Bonn, 53,127 Bonn, Germany; Frank Anton Giordano: Department of Radiation Oncology, University Medical Center Mannheim, 68,167 Mannheim, Germany and DKFZ-Hector Cancer Institute, University Medical Center Mannheim, 68,167 Mannheim, Germany; Matthias Bernhard Stope: Department of Gynaecology and Gynaecological Oncology, Physical Plasma Laboratories, University Hospital Bonn, 53,127 Bonn, Germany; Eleni Gkika: Department of Radiation Oncology, University Hospital Bonn, 53,127 Bonn, Germany; Leonard Christopher Schmeel: Department of Radiation Oncology, University Hospital Bonn, 53,127 Bonn, Germany.
Name and Contact Information for the Trial Sponsor {5b}	Department of Radiation Oncology University Hospital Bonn Venusberg-Campus 1 53,127 Bonn Germany
Role of Sponsor {5c}	The sponsor and funding body have no role in the design of the study, in collection, analysis, and interpretation of the data or in the writing of the manuscript.

## Introduction

### Background and rationale {6a}

Early breast cancer remains the most common oncological diagnosis [1]. Treatment usually involves lumpectomy followed by adjuvant breast irradiation to improve local control and (in most cases) survival [2]. Radiation dermatitis (RD), characterised by erythema, pruritus, discomfort, and dry or moist desquamation, is the most common acute side effect of breast irradiation, occurring in up to 85% of patients [3, 4]. Although severe cases have become rare following advancements in radiation treatment technique and the exploration of new fractionation regimens, even moderate or mild symptoms are known to impact quality of life and self-image [5, 6]. Potent preventative and therapeutic options remain limited and due to insufficient and sometimes even conflicting evidence, recommendations can currently only be made for a handful of interventions, resulting in substantial variations in RD management amongst practitioners and institutions [7–11].

Non-invasive physical plasma (NIPP) is an effective treatment modality for several skin conditions, including psoriasis, eczema, diabetic ulcers, and different types of dermatitis [12, 13]. Physical plasma can be regarded as the fourth state of matter and is a highly energetic, partially ionised gas characterised by free electrons [14, 15]. NIPP is created using a high-frequency alternating field under atmospheric pressure, therefore only reaching room temperatures and making it safe for clinical application. Dielectric barrier discharges (DBDs) are a type of NIPP, generated out of ambient air, omitting the need for a carrier gas [16]. Several trials have demonstrated that this reactive mix of electrons, ions, excited atoms, and reactive oxygen and nitrogen species (RONS) positively affects tissue healing in a dose-dependent manner [17]. NIPP treatment is well-tolerated with multiple trials confirming its safety and thus far complete absence of side effects [13, 18–20].

Recent evidence has identified the skin microbiome as a risk factor for (severe) RD, postulating the hypothesis that bacterial decolonisation might be an effective prophylactic approach, which would be the main mode of action of NIPP in this context [21–23]. Furthermore, NIPP promotes proliferation and migration of keratinocytes, fibroblasts, and endothelial cells, thus facilitating tissue regeneration [24]. The expression of wound healing gene signatures alongside significant changes to the human skin barrier lipid stoichiometry are two additional modes of action, which are thought to positively impact the complex pathophysiology of RD [25, 26]. In an investigator-initiated first-in-human phase 1 benchmarking trial (*n* = 30), we previously reported safety and feasibility features of a topical DBD-generated NIPP-based prevention of acute RD [27]. In the follow-up phase 2 trial (*n* = 64), we established the preliminary efficacy of this approach, with a positive impact on both clinician- and patient-reported endpoints [28]. As expected, tolerability of NIPP was excellent and no side effects occurred.

### Objectives {7} and trial design {8}

In this current prospective phase 3 randomised double-blind placebo-controlled trial, we aim to establish the effects of add-on NIPP on the incidence and severity of RD and associated symptoms in patients undergoing breast irradiation, with clinician-assessed RD (as per CTCAE v5.0) upon radiation treatment completion as the primary endpoint.

## Methods

### Study setting {9}

Patients will be recruited by the Department of Radiation Oncology at the University Hospital Bonn in Germany, upon referral from the Department of Gynaecology and Gynaecological Oncology, Division of Senology, after multidisciplinary recommendation of adjuvant breast irradiation in the joint interdisciplinary tumour board.

### Eligibility criteria {10}

#### Inclusion criteria

Strict inclusion and exclusion criteria are defined to ensure a homogeneous patient collective. All patients with invasive breast cancer (ICD-10 C50.9) or ductal carcinoma in situ (ICD-10 D05.10) scheduled for adjuvant breast irradiation following breast-conserving surgery (i.e. lumpectomy) will be screened. The following inclusion criteria apply:

* Age ≥ 18 years (no upper limit);
* Moderately hypofractionated radiation regimen in 15 fractions, with at least a whole-breast target volume and with or without a boost to the tumour bed (either simultaneous integrated or sequential);
* Oral and written informed consent.

#### Exclusion criteria

To omit bias in RD development and grading, subjects will not be included if any of the following exclusion criteria applies:

* Synchronous metastatic disease;
* Mastectomy;
* Reconstruction with breast implant;
* Prior skin infiltration by tumour necessitating the use of a bolus;
* Impaired surgical wound healing at the time of radiation treatment initiation;
* Alternative fractionation regimens;
* History of ipsilateral breast irradiation;
* Any pre-existing dermatological disorder;
* Active dermatitis;
* Current treatment with topical or oral corticosteroids;
* Patient refusal to participate;
* Subjects without legal capacity to understand the nature, scope, significance, or consequences of a clinical trial.

### Informed consent {26a} {26b}

Informed consent will be obtained by specifically trained radiation oncologists registered as investigators for the trial.

## Interventions

### Explanation for the choice of comparators {6b}

Both the previous phase 1 and 2 trial have demonstrated preliminary efficacy of NIPP in the context of RD prevention during breast irradiation [27, 28]. NIPP will be investigated as an add-on modality to standard skin care with a moisturising lotion.

### Intervention description {11a}

#### Radiation treatment

All patients will receive 36 − 40.05 Gy in 15 fractions of 2.4 − 2.67 Gy each to the whole breast and 40.05 Gy in 15 fractions of 2.67 Gy each to the partial breast. Patients ≤ 50 years or those with established risk factors (≥ pT2, HER2 positive, triple-negative, or poor cell differentiation) will receive either a simultaneous integrated (48 Gy in 15 fractions of 3.2 Gy each) or sequential boost (10 − 16 Gy in 5 − 8 fractions of 2 Gy each) to the tumour bed (choice at the discretion of the treating radiation oncologist and patient). Target volume delineation will follow international standards [29–31]: the whole-breast clinical target volume (CTV) will be defined as all residual ipsilateral glandular breast tissue, whereas the partial-breast CTV will be delineated as the lumpectomy cavity (including surgical clips and postoperative seroma), radially expanded by 1 − 1.5 cm. Both CTV and planning target volume (PTV) will be cropped 5 mm from the skin surface. Treatment technique will be photon-based 6 MV sliding window intensity-modulated radiotherapy (IMRT), hybrid 6 and 10 MV volumetric modulated (partial) arc therapy, or electronic tissue compensation (eComp). Planning will aim for a homogeneous dose distribution while avoiding hotspots in the skin region and following the International Commission on Radiation Units and Measurements (ICRU) recommendations for dose limits of 95–107%. All patients will be treated on a TrueBeam STx (Varian Medical Systems, Palo Alto, CA, USA) linear accelerator in a supine position on a breast board. Left-sided breast irradiation will be performed in deep inspiration breath-hold (DIBH) for compliant patients. The irradiated breast skin will be defined as the 5 mm layer between the body contour and the radially expanded (1.5 cm) whole-breast PTV. Breast skin dose will be assessed dosimetrically (e.g. V_107%_) to ensure balance between groups.

#### Standard skin care

Institutional standard skin care with urea-based lotion (UreaRepair PLUS 5%, Eucerin, Beiersdorf, Hamburg, Germany) will be applied twice daily to the whole breast from the first day of treatment onwards until 4 weeks after radiotherapy completion. All patients will be provided with oral and written instructions alongside information on general skin care during radiotherapy (e.g. washing with mild soap, drying with a soft towel, deodorant use).

#### Non-invasive physical plasma

**Fig. 1:**
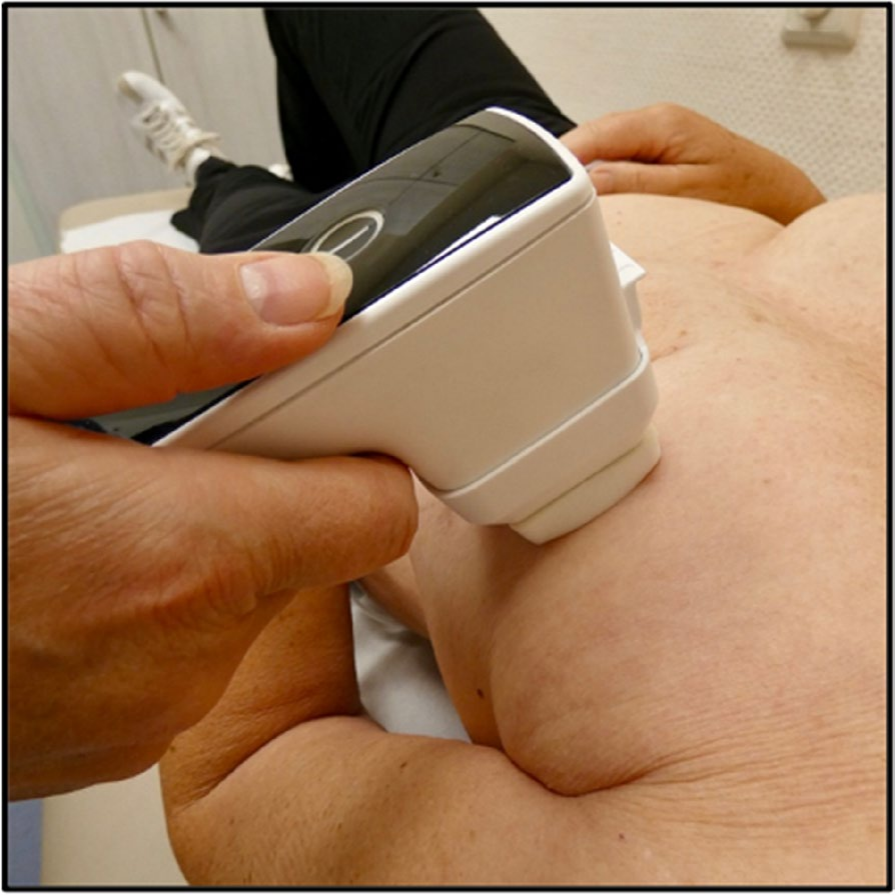
Generation and application of NIPP on the breast skin. The device is pressed loosely on the skin with a separate 4 × 4 cm spacer for each patient and each visit. NIPP = non-invasive physical plasma. Image from Dejonckheere et al. [27], with permission

Several NIPP-generating devices are commercially available. The device used in this trial will be plasma care (terraplasma medical, Garching, Germany). This wireless, handheld, class 1 medical device is certified for use in the context of dermatological disorders, specifically for prophylaxis and therapy of inflammatory skin diseases caused by ionising radiation. In this setting, it can also explicitly be used as an add-on to standard skin care. The device is used with a separate sterile spacer with a 4 × 4 cm NIPP contact surface. For each patient and each visit, the device is then pressed loosely onto the patient’s breast skin, ensuring that the distance to the skin is always kept the same (Fig. 1). The application will be repeated in each of the breast quadrants. Based on the initial feasibility and dose-escalation trial, the daily treatment time is set at 120 s, using the device’s preset programme to ensure a constant dose of NIPP [27]. NIPP will be applied daily by a trained radiation oncology nurse, following every radiation fraction.

#### Placebo

Patients randomised to the control arm will receive sham treatment with an identical device, which produces the same sound and feeling, yet does not produce NIPP. To keep the devices separated, they will be marked “1” and “2”. The treating radiation oncology nurse is the only one who will be aware of the exact allocation.

### Criteria for discontinuing or modifying allocated interventions {11b}

At the discretion of the investigators, patients presenting with grade ≥ 2 RD, moist desquamation, or intense pain can be prescribed topical corticosteroids (methylprednisolone aceponate) until symptoms resolve. Any medication prescribed for local symptoms will be documented regarding dose, duration, and mode of application. Patients can withdraw informed consent at any point in time. If so, the reason(s) will be documented. Cross-over between arms is not planned.

### Strategies to improve adherence to interventions {11c}

Compliance with standard skin care will be checked by the treating radiation oncologist during the scheduled patient visits.

### Relevant concomitant care permitted or prohibited in the trial {11d}

Patients will be encouraged not to use any complementary topical treatments on the breast skin for the duration of the trial. Other radiotherapy-related acute toxicities will be treated *lege artis*.

### Provisions for post-trial care {30}

All patients will receive standard follow-up. Due to the excellent safety profile of NIPP, harm from trial participation is not expected.

### Outcomes {12}

#### Clinician-reported outcome (CRO)

Patients will be evaluated during weekly on-treatment visits. Any additional visits requested by the participants will also be documented. RD severity of the irradiated breast will be assessed according to the National Cancer Institute’s Common Terminology Criteria for Adverse Events (CTCAE; version 5.0) [32]. Furthermore, hyperpigmentation, breast oedema, and desquamation (dry or moist) will be assessed and recorded. Upon radiation treatment completion, as well as 2 and 6 weeks later, acute toxicity will be reassessed. An experienced breast radiation oncologist, blinded to the allocation, will perform all clinical assessments. The topical corticosteroid prescription rate (to alleviate radiation dermatitis-related symptoms) will also be assessed in both arms.

#### Patient-reported outcome (PRO)

At the end of the radiation course and during the two follow-up visits, the patient-assessed modified Radiation-Induced Skin Reaction Assessment Scale (RISRAS) will be recorded [33]. All patients will report their maximum breast-related experience of pain, itching, burning, and limitations in daily activities. Items are scored on a 4-point Likert scale: 0 = not at all; 1 = a little; 2 = quite a bit; 3 = very much.

#### Objective assessment

At baseline, during every visit, on the last day of radiation treatment, as well as during both follow-up visits, four skin colour readings will be performed across the irradiated breast (one in each of the breast quadrants) with a reflectance spectrophotometer (CR-10 Plus, Konica Minolta, Tokyo, Japan). This compact device has previously been validated to objectively assess RD in a non-invasive manner [34, 35]. The automatically performed measurements are based on the Commission Internationale de l’Éclairage (CIE) system of tristimulus values. They describe a measured colour in three coordinates using the L*a*b* system: lower L* values describe darker skin (hyperpigmentation) and higher a* values indicate redness or erythema (RD). The b* values describe the position on a scale from blue to yellow and are of secondary importance in the acute setting.

#### Patient-reported experience (PRE)

During the last follow-up visit, patient-reported experiences will be captured through an exit questionnaire with several yes–no statements. To further enhance public and patient involvement, this questionnaire was adapted from the one used in the phase 1 and 2 trial, further refined based on included patient’s feedback.

### Participant timeline {13}

**Fig. 2:**
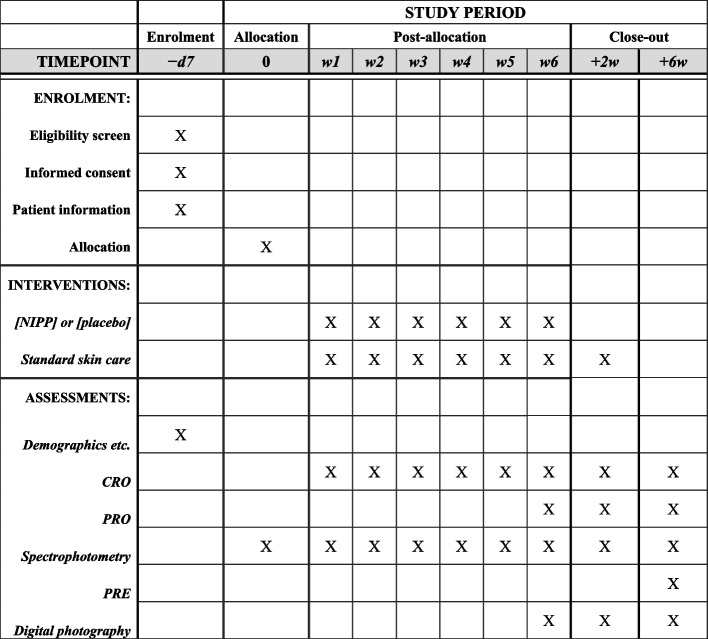
Participant timeline. d = day; w = week; NIPP = non-invasive physical plasma; CRO = clinician-reported outcome; PRO = patient-reported outcome; PRE = patient-reported experience

The participant timeline is shown in Fig. 2.

### Sample size {14}

The primary endpoint of this trial is clinician-assessed RD (as per CTCAE v5.0) in the NIPP and placebo group upon radiation treatment completion. The sample size calculation is based on the results of our phase 2 trial [28]. A decrease in mean clinician-reported RD severity upon radiation treatment completion of at least one third will be assumed with NIPP (RD severity with standard skin care: 1.06 ± 0.71 [4]). To detect such a difference (*Δ* = 0.35; standard deviation [SD] = 0.7) with 80% statistical power with a two-tailed *t*-test at a significance level of 5%, an analysed sample size of *n* = 128 is needed (i.e. 64 patients per treatment arm). We plan to analyse the CTCAE severity grades as ordinal data using a non-parametric Mann–Whitney-Wilcoxon test. The required sample size is, however, computed for a parametric test. In order to achieve the same degree of confidence as a similar parametric test, a larger sample size is thus needed for the non-parametric test [36]. As a rule of thumb, sample size will therefore be increased by an additional 10%, resulting in *n* = 140 (i.e. 70 patients per treatment arm). Based on both previous trials, we expect 20% drop-out after initial informed consent (*n* = 168 randomised). Of all patients being referred to our department, we expect 20% meeting inclusion criteria but declining participation (*n* = 202 enrolled and assessed for eligibility).

### Recruitment {15}

Based on both previous trials, we expect completion of the trial within 36 months from initiation to be feasible.

#### Assignment of interventions: allocation

### Sequence generation {16a}

Using intrapatient randomisation (the irradiated breast is divided into a lateral and medial half; one breast half receives experimental treatment, while the other breast half receives standard of care or placebo) has proven useful in several trials investigating (acute or chronic) RD management approaches [37–40]. This omits the need for stratification based on known risk factors influencing the RD risk and limits confounding when assessing trial endpoints. The volatile nature of NIPP, however, does not allow such trial design, as potential diffusion to the contralateral (placebo) breast half with a subsequent effect is likely, as was the case in our phase 2 trial [28]. Therefore, a separate control group receiving standard skin care + placebo will be recruited. A 1:1 randomisation (NIPP:placebo) will be undertaken, with patient stratification based on two proven risk factors for RD development and severity: total radiation dose (i.e. presence or absence of a boost to the tumour bed) and irradiated breast volume (as measured in the radiation treatment planning computer tomography: small < 400 mL, medium 400 − 800 mL, large > 800 mL) [35, 41]. Patients will be assigned to intervention or placebo using a stratified block randomisation process (block size will be disclosed upon final publication of the results), using a computer-generated random number list, created by a team member not involved in the clinical part of the trial. Other factors which are known to modulate the RD risk (e.g. body mass index, diabetes mellitus, smoking history, Fitzpatrick skin type) will not be included in the stratification process, but will be documented and presented (including balance between groups).

### Concealment mechanism and implementation {16b} {16bc}

Allocation will be undertaken by a trained radiation oncology nurse who is also the NIPP/placebo device operator. Allocation information will be stored on a password-protected secure server, inaccessible to the blinded investigators.

## Assignment of interventions: blinding

### Who will be blinded? {17a}

Patients and treating physicians will be blinded until completion of the trial for individual patients; only the device operator is aware of the respective allocation and will not be conducting any assessments. Data analysts and statisticians will also be blinded.

### Procedure for unblinding {17b}

Upon trial completion for individual participants, unblinding is performed by the treating radiation oncology nurse, if requested by the patient. Sealed emergency envelopes will be stored at a location known and accessible to all trial personnel and can be used for premature unblinding in emergency situations.

## Data collection and management

### Plans for assessment and collection of outcomes {18a}

The following data will be recorded upon inclusion in the trial: demographics, medical history, tumour characteristics, treatment characteristics, and dosimetric parameters. During radiation treatment and follow-up, acute toxicity will be assessed as mentioned above (CRO, PRO, objective assessment, and PRE).

### Plans to promote participant retention and complete follow-up {18b}

Patients are seen daily during radiation treatment. Upon withdrawal of informed consent, the reason will be recorded and data available until this point in time will be used for analysis.

### Data management {19}

All data recorded during the trial will be pseudonymised, with patients being identifiable by a unique patient number. The identification list will be stored on a password-protected secure server. Data will be collected on trial-specific documentation sheets, signed and dated by the assessing radiation oncologist for every separate patient visit. All data will be transferred into a single database (electronic case report form). All trial documents will be securely stored for 10 years.

### Confidentiality {27}

All data will be handled according to the General Data Protection Regulation (GDPR), ensuring strict confidentiality.

### Biological specimens {33}

Not applicable.

#### Statistical methods

### Statistical methods for primary and secondary outcomes {20a}

Mean, median, SD, and range will be calculated for all applicable clinical data. Differences in baseline patient characteristics between both treatment arms will be assessed with Pearson’s *χ*^*2*^ or Student’s unpaired *t* test, as appropriate. The primary endpoint will be assessed using the Mann–Whitney-Wilcoxon test, and an odds ratio (with a 95% confidence interval) will be calculated for the occurrence of grade ≥ 2 RD. For the pairwise comparison of categorical variables (clinician- and patient-reported outcomes) between treatment groups, the Mann–Whitney-Wilcoxon test will be performed. The comparison of continuous variables (spectrophotometric readings) will be performed with Student’s unpaired *t* test (after proving homoscedasticity with Levene’s test). The statistical significance level will be defined as *p* < 0.05.

### Interim analyses {21b}

There are no planned interim analyses. The excellent safety profile of NIPP renders an interim safety analysis or stopping rules obsolete. The efficacy of the intervention versus placebo will only be assessed if the preplanned sample size has been reached.

### Methods for additional analyses {20b}

Not applicable.

### Methods in analysis to handle protocol non-adherence {20c}

All analyses will be modified intention-to-treat, excluding participants not initiating allocated treatment. A secondary per protocol analysis will be performed as well.

### Plans to give access to the full protocol, participant-level data, and statistical code {31c}

Not applicable.

#### Oversight and monitoring

### Composition of the coordinating center and trial steering committee {5d}

The coordinating center consists of the principal investigator, investigators (all with full delegation), and the involved study nurses. Investigators will recruit and enroll patients in addition to performing the blinded outcome assessments. The unblinded study nurses will perform the administration of the allocated treatment. Five prescheduled investigator meetings are planned during the recruitment period to evaluate progress of the trial or assess any issues that might have arisen.

### Composition of the data monitoring committee {21a}

Data management and monitoring will be performed by a clinical research associate of the Studienzentrale Bonn (SZB), following the monitoring manual designed for this trial, to ensure reliability of trial results.

### Adverse event reporting and harms {22}

Not applicable. In both initial trials, no adverse events were reported, in line with previous trials using NIPP for other skin disorders, confirming the complete absence of side effects.

### Frequency and plans for auditing trial conduct {23}

Yearly audits (on-site monitoring) will be carried out by a clinical research associate of the SZB, to ensure rights and safety of included patients and assess the reliability of trial results. Independent monitoring will be performed in accordance with a trial-specific risk-adapted manual, signed by all parties involved in conducting this trial, and involves the project management group and all involved trial personnel. Upon trial completion by the last patient, a close-out visit is scheduled.

### Plans for communicating important protocol amendments to relevant {25}

Amendments to the trial protocol can only be implemented upon approval by the responsible Institutional Review Board, through the principal investigator of the trial. Any changes to the protocol will be communicated with the funder and all trial personnel. A copy of the revised protocol will be added to the Investigator Site File and training will be provided if needed. The protocol in the clinical trial registry will be updated accordingly. In general, any deviations from the trial protocol will be documented using a breach report form.

### Dissemination plans {31a}

Results will be published in a peer-reviewed journal after database clearance and subsequent closure by a clinical research associate of the SZB.

## Discussion

RD remains the most common acute side effect of breast irradiation and radiotherapy in general. Progress in the development of new prophylactic and therapeutic agents has been slow, leading to a considerable physical and psychological impact on numerous patients. While topical corticosteroids are effective in reducing RD-related symptoms, their widespread and prolonged use remains limited due to the associated side effect profile [42, 43]. Care should be taken especially if moist desquamation is present, as topical corticosteroids might delay wound healing or even promote infection.

Trials investigating new ways to prevent or treat RD often recruit heterogeneous patient collectives in terms of dose-fractionation regimens and treatment sites, with the majority lacking a uniform control group or adequate placebo control. Another issue is the subjectivity of physician-assessed RD gradings such as CTCAE or RTOG, the most common primary endpoints, with considerable inter- and intra-observer variability and significant discrepancies with the patient’s perspective [44]. To accurately investigate prevention and treatment strategies, there is a need for sufficiently powered randomised controlled trial, recruiting a homogeneous patient collective in terms of RD risk, with a robust design (including CRO, PRO, and objective assessments) and apt placebo control, as is the case in this proposed trial protocol.

If the trial results in breast cancer patients are promising, the beneficial effects and lack of side effects of NIPP could potentially be translated to other patients and treatment sites at-risk for developing RD, e.g. those receiving radiotherapy for head and neck or skin cancer, or even accidental radiation injuries of the skin [45]. Adjuvant breast irradiation is chosen as the primary setting to investigate the use of NIPP in this trial due to its high prevalence, homogeneous treatment sites and radiation dose-fractionation concepts.

### Trial status

The current version of the protocol is version 1.0, dated on the 9th of November 2023. The trial has started enrolment in the first quarter of 2024 and the first patient was randomised on the 15th of February 2024. The trial is scheduled to take 36 months until completion (until the last quarter of 2026).

## Data Availability

Results will be published in a peer-reviewed journal upon clearance and closure of the database. Dissemination of individual patient data is not planned.

## Abbreviations

CIE: Commission Internationale de l’Éclairage
CRO: Clinician-reported outcome
CTCAE: Common Terminology Criteria for Adverse Events
CTV: Clinical target volume
DBD: Dielectric barrier discharges
DIBH: Deep inspiration breath-hold
eComp: Electronic tissue compensation
GDPR: General Data Protection Regulation
Gy: Grey
HER2: Human epidermal growth factor receptor 2
ICD: International Classification of Diseases
ICRU: International Commission on Radiation Units and Measurements
IMRT: Intensity-modulated radiotherapy
MV: Megavolt
NIPP: Non-invasive physical plasma
PRE: Patient-reported experience
PRO: Patient-reported outcome
PTV: Planning target volume
pT2: Primary tumour > 20 mm
RD: Radiation dermatitis
RISRAS: Radiation-Induced Skin Reaction Assessment Scale
RONS: Reactive oxygen and nitrogen species
RTOG: Radiation Therapy Oncology Group
SD: Standard deviation
SZB: Studienzentrale Bonn
